# Lower lip as a photoacoustic imaging window for detection of CTC clusters: A feasibility phantom study^☆^

**DOI:** 10.1016/j.pacs.2026.100896

**Published:** 2026-10-08

**Authors:** Ashkan Ghanbarzadeh-Dagheyan, Elahe Rastegar Pashaki, Kirsten Pondman

**Affiliations:** a Biomedical Photonic Imaging Group, TechMed Centre, University of Twente, Enschede, The Netherlands; b Physics of Fluids, TechMed Centre, University of Twente, Enschede, The Netherlands; c BIOS Lab-on-a-chip Group, MESA+ Institute for Nanotechnology, TechMed Centre, University of Twente, Enschede, The Netherlands

**Keywords:** Photoacoustic imaging, Circulating tumor cells, Melanoma, Singular value decomposition, Kymograph analysis, Lower lip phantom

## Abstract

Detection and continuous monitoring of circulating tumor cells (CTCs) using photoacoustic imaging remains challenging because of weak transient signals and strong blood-background absorption. This proof-of-concept study investigated the lower lip as a potential photoacoustic imaging window for melanoma CTC detection. A tissue-mimicking lower-lip phantom incorporating vessel-mimicking channels, physiologically relevant flow conditions, and donated human blood was developed. Melanoma-cell targets, including HS936.T and melanin-enhanced Mel624+α-MSH clusters, were imaged using photoacoustic tomography. Singular value decomposition (SVD)-based background suppression and kymograph-based temporal analysis were applied to enhance and characterize transient target-passage events. A composite kymograph-derived Target Score was developed for both automated classification and temporal guidance of visual inspection. Detectability depended on target size, melanin content, and surrounding medium. Mel624+α-MSH clusters remained detectable under blood-containing conditions, whereas similarly sized HS936.T clusters were not detected. Single HS936.T cells were also not detected, and the smallest detected cluster at the tested melanin concentrations was 500μm. Operator-referenced automated classification achieved an F1 score of 0.800 and an AUC of 0.733, while temporal guidance captured 83.3% of visually identified passages and reduced the temporal search space by 91.79%. Limited blood and cell availability constrained systematic evaluation and replication. At this proof-of-concept stage, the framework provides a semi-automated approach combining automatic scoring with focused visual inspection. These findings support further investigation of the lower lip as a potential site for photoacoustic monitoring of circulating melanoma cells and provide a foundation for developing automated and semi-automated CTC detection approaches in future studies.

## 1 Introduction

Metastasis is responsible for the vast majority of cancer-related deaths, and circulating tumor cells (CTCs) are widely regarded as key mediators and biomarkers of this process [1], [2]. Because CTCs are shed from primary or metastatic lesions into the bloodstream, their detection can provide valuable information for early diagnosis, prognosis, and treatment monitoring. Even in metastatic disease, CTCs are extremely rare, with reported concentrations on the order of 1–10 cells per milliliter of blood, compared to millions of leukocytes and billions of erythrocytes [3]. Current clinical methods for CTC analysis, such as CellSearch and other *ex-vivo* enrichment approaches, are based on small blood samples and therefore interrogate only a minute fraction of the total circulating blood volume [2], [4], [5]. This sparse sampling is particularly problematic given the rarity and temporal variability of CTCs in circulation, and it limits the ability of existing technologies to provide continuous monitoring.

Photoacoustic imaging offers an attractive route toward continuous, non-invasive CTC monitoring by combining optical absorption contrast with ultrasonic detection depth [6]. Following absorption of a short laser pulse, a local pressure rise is generated through thermoelastic expansion. Under stress and thermal confinement, the initial pressure can be written as

$$p_{0}=\Gamma \mu_{a}F,$$

where Γ is the Grüneisen parameter, $\mu_{a}$ is the optical absorption coefficient, and F is the local optical fluence [6], [7]. In biological tissue, this mechanism enables ultrasound-resolution imaging with contrast governed by optical absorption rather than acoustic impedance alone, providing a major advantage over conventional ultrasound for detecting strongly absorbing targets such as melanin-containing melanoma cells [6], [7]. Recent advances in photoacoustic microvascular imaging have further highlighted its capability to resolve complex, dynamic physiological phenomena, such as blood–brain barrier homeostasis and deep-tissue microvascular dynamics *in vivo* [8], [9]. Prior studies have already demonstrated the promise of photoacoustic flow cytometry for detecting circulating melanoma cells and other rare targets *in vivo* [2], [10], [11].

However, a major unresolved challenge is the identification of anatomical sites that simultaneously offer low optical attenuation, low acoustic attenuation, and practical access for repeated or continuous monitoring. In this context, the lower (or upper) lip is a promising but largely unexplored anatomical window for photoacoustic CTC detection. The oral mucosa of the inner lower lip contains superficial blood vessels at shallow depths and exhibits lower attenuation than keratinized skin. Therefore, it may facilitate both label-free detection of intrinsically absorbing targets and higher-sensitivity contrast-enhanced photoacoustic detection. Importantly, the lower lip has already been shown to support non-invasive optical microscopy of blood cells in vivo; Winer et al. demonstrated imaging of leukocytes in vessels located approximately 50–150μm below the mucosal surface in the inner lower lip [12]. In parallel, ultrasound-based work has suggested that the oral mucosa can serve as a suitable site for intravascular cell detection, and specifically proposed this region as a favorable target for in vivo monitoring [13]. Assuming that CTCs circulate systemically throughout the body, the lower lip may provide a convenient optical–acoustic access point for repeated surveillance. The same reasoning goes for the upper lip. This non-invasive labial framework aligns closely with broader clinical translation efforts that focus on scaling photoacoustic microscopy and wearable sensors from benchtop systems into practical patient-monitoring tools [14], [15]. In the longer term, this concept could motivate wearable implementations, for example a mouthguard-integrated device capable of monitoring labial vessels during sleep or during treatment in patients at high risk of metastasis (Fig. 1). Such continuous monitoring could contribute to development of preventive measures against metastasis and optimized treatment plans informed by CTC activity.

**Fig. 1:**
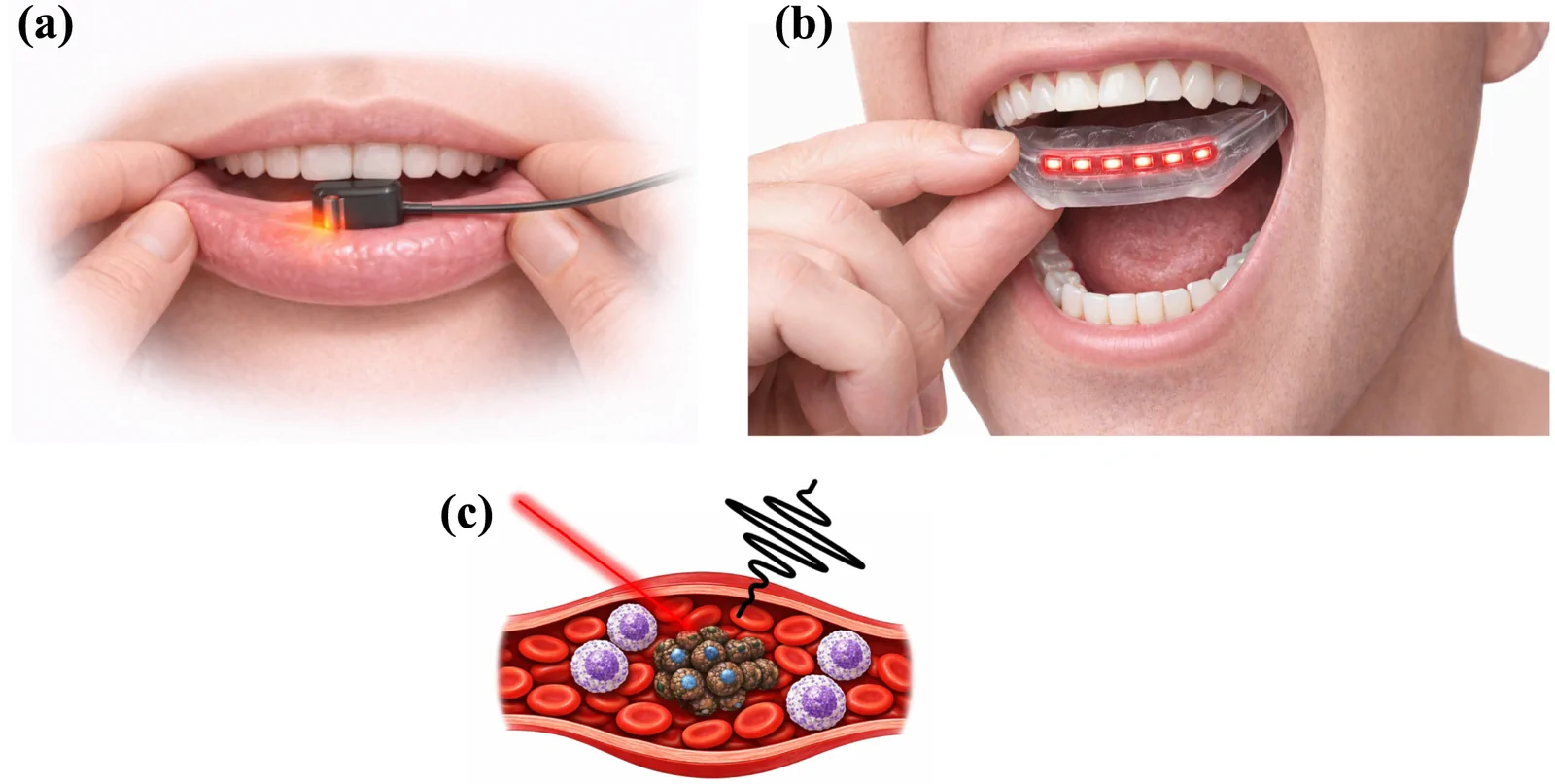
Lower lip as a potential location for low-attenuation photoacoustic (PA) sensing of healthy and cancerous blood cells. (a) Vision of a pre-clinical photoacoustic sensor capable of detecting circulating tumor cells (CTCs); (b) a wearable version of a night mouthguard capable of monitoring blood for CTCs in the upper or lower lip over a long period during sleep; (c) schematic of PA imaging/sensing of the contents of a small artery in the lower lip (e.g., inferior labial artery). (The figure was generated using ChatGPT (OpenAI) as an AI-assisted conceptual illustration. No third-party imagery was adapted or reproduced.)

In this study, we investigate the feasibility of this concept using a realistic *in-vitro* phantom of the lower lip vasculature. The phantom was designed to approximate the geometry and relevant physical properties of inferior labial tissue, and physiologically relevant flow conditions were implemented. Experiments were conducted using donated human blood to provide a physiologically relevant optical absorption background and to assess detection performance under realistic conditions. In addition, human melanoma cell lines were cultured and used as CTC models with varying pigmentation characteristics. For this purpose Mel624 and HS936.T were selected to represent differing pigmentation states [16]. A similar approach may be applied to labeled targets and other endogenous or exogenous chromophores. Using photoacoustic tomography, we evaluate whether the lower lip can function as a practical photoacoustic window for intravascular detection of melanoma CTC-like targets. As a feasibility study, the present work establishes an initial experimental framework for evaluating photoacoustic detection of melanoma cell clusters under controlled flow conditions. By combining a physiologically relevant blood background, melanoma cell models with distinct pigmentation characteristics, and quantitative image-based detection, the study provides insight into the factors governing target visibility and detection in the lower-lip geometry. These findings characterize both the capabilities and current boundaries of the approach and provide a foundation for further development toward *in-vivo* and wearable photoacoustic monitoring strategies in future.

## 2 Materials and methods

This section describes the experimental framework used to evaluate the feasibility of photoacoustic detection of melanoma CTC-like targets in a lower lip model. First, the in-house photoacoustic imaging system and data acquisition approach are presented. Next, the development of the tissue-mimicking phantom and the flow setup designed to approximate physiological conditions in the inferior labial vasculature are described. Finally, the preparation of melanoma cell models and blood-based experiments are detailed.

### 2.1 Photoacoustic imaging system

Photoacoustic imaging was performed using an in-house developed photoacoustic-ultrasound (PA-US) system consisting of a pulsed laser (PhotoSonus X, EKSPLA, Lithuania) and a programmable ultrasound platform (Vantage 256, Verasonics, USA). Acoustic detection was achieved using a linear array transducer (L11-4v, Verasonics), which was used in receive mode for photoacoustic signal acquisition. The laser operated at a wavelength of 700 nm with a pulse repetition rate (PRR) of 100 Hz and a pulse duration of 2–5 ns. Optical illumination was delivered through a linear array of optical fibers positioned alongside the ultrasound probe to illuminate the imaging region. (Fig. 2-(a) and -(c)). The ultrasound system was synchronized with the laser to ensure accurate acquisition of photoacoustic signals.

The L11-4v transducer had a center frequency of 7.5 MHz and a nominal fractional bandwidth of approximately 91% at the −6 dB level, based on published characterization of the transducer [17]. Photo-acoustic receive data were sampled at 30 MHz using the Vantage 256 system, corresponding to four times the transducer center frequency. The default Verasonics receive settings and gain were used, without additional user-applied signal amplification. Photoacoustic images were reconstructed using delay-and-sum (DAS) beamforming within the Verasonics MATLAB framework. The reconstruction was based on the manufacturer-provided SetUpL11_4vFlashAngPA.m example and the photoacoustic acquisition and reconstruction procedure described by Kratkiewicz et al. [18].

The optical illumination was characterized by an approximately 10 cm^2^ illumination area. The pulse energy was measured at the input of the optical fiber after the optical attenuation stage, and the corresponding fluence was calculated using the illumination area at the fiber output. At 700 nm, the maximum measured pulse energy was approximately 24.2 mJ, corresponding to a maximum fluence of 2.42 mJ/cm^2^. A conservative 100% fiber-coupling assumption was used for the fluence and safety assessment; therefore, the actual optical energy reaching the sample was expected to be lower.

During the experiments, the live photoacoustic acquisition display was recorded as movies at approximately 30 frames/s for subsequent review and analysis. This recording frame rate was determined by the software used to capture the live acquisition display and should not be interpreted as the underlying photoacoustic pulse-acquisition rate. The laser PRR was 100 Hz and therefore determined the temporal sampling of the photoacoustic excitation sequence (see Table 1).

**Table 1:** Summary of photoacoustic acquisition, illumination, and image-reconstruction parameters.

Parameter	Value/Description
Laser	PhotoSonus X (EKSPLA, Lithuania)
Excitation wavelength	700 nm
Pulse repetition rate	100 Hz
Pulse duration	2–5 ns
Illumination geometry	Linear array of optical fibers positioned alongside the L11-4v transducer
Optical excitation beam width (effective)	≈20 mm
Illumination area	≈10 cm^2^
Maximum measured pulse energy	≈24.2 mJ at 700 nm
Maximum calculated fluence	≈2.42 mJ/cm^2^ at 700 nm
Fiber coupling assumption	100% for conservative fluence/safety assessment
Transducer	L11-4v linear array (Verasonics)
Center frequency	7.5 MHz
Nominal −6 dB fractional bandwidth	≈91%
Receive system	Vantage 256 (Verasonics)
Sampling frequency	30 MHz
Receive gain	Default Verasonics setting
Image reconstruction	Delay-and-sum (DAS) beamforming
Recorded movie frame rate	≈30 frames/s

### 2.2 Data acquisition and processing

#### 2.2.1 SVD filtering

Photoacoustic data were recorded as image sequences. Due to the presence of strong stationary background signals and system-related artifacts, individual frames did not always allow reliable visual identification of moving targets. To enhance the visibility of flowing particles and cells, a spatiotemporal filtering approach based on singular value decomposition (SVD) was applied to the recorded image sequences. SVD-based clutter suppression has also been used for separating moving contrast-agent signals from tissue clutter in high-frame-rate ultrasound particle image velocimetry (PIV), including in patient arterial measurements [19]. Within tissue, especially when deeper vessels are targeted, a high level of clutter often appears in ultrasound frames; therefore, SVD can be essential for separating contrast agents from the background and achieving reliable PIV results.

For the present analysis, the first two dominant singular components were removed consistently in all datasets. The cutoff was selected empirically by inspection of the reconstructed data, with the objective of suppressing the predominantly static background while retaining temporally varying moving-object signals. The resulting enhancement of moving objects is illustrated in Fig. 3-(d). Slope-based analysis of the eigenvalue decay has also been reported for selecting the clutter rank in SVD-based ultrasound microvascular imaging [20], which can be utilized as an alternative method for dominant singular component selection.

A broad manually defined region of interest (ROI) was used for computational efficiency. The ROI spanned the full width of the movie frame and extended vertically to encompass the complete vessel lumen together with a small amount of tissue-mimicking material above and below the lumen. The surrounding tissue-mimicking phantom produced essentially no non-static signal in the acquisition. The ROI was therefore selected primarily to reduce computational time and memory requirements and was kept consistent across datasets.

After filtering, residual clutter signals remained present, particularly in frames without target passage. Identification of melanoma cell-like targets was based on spatially localized signals that persisted across consecutive frames and exhibited coherent motion along the vessel, in contrast to stochastic and temporally inconsistent background fluctuations. The detection approach relies on the strong optical absorption of melanin present in melanoma cells, enabling their identification as localized photoacoustic sources [21].

**Fig. 2:**
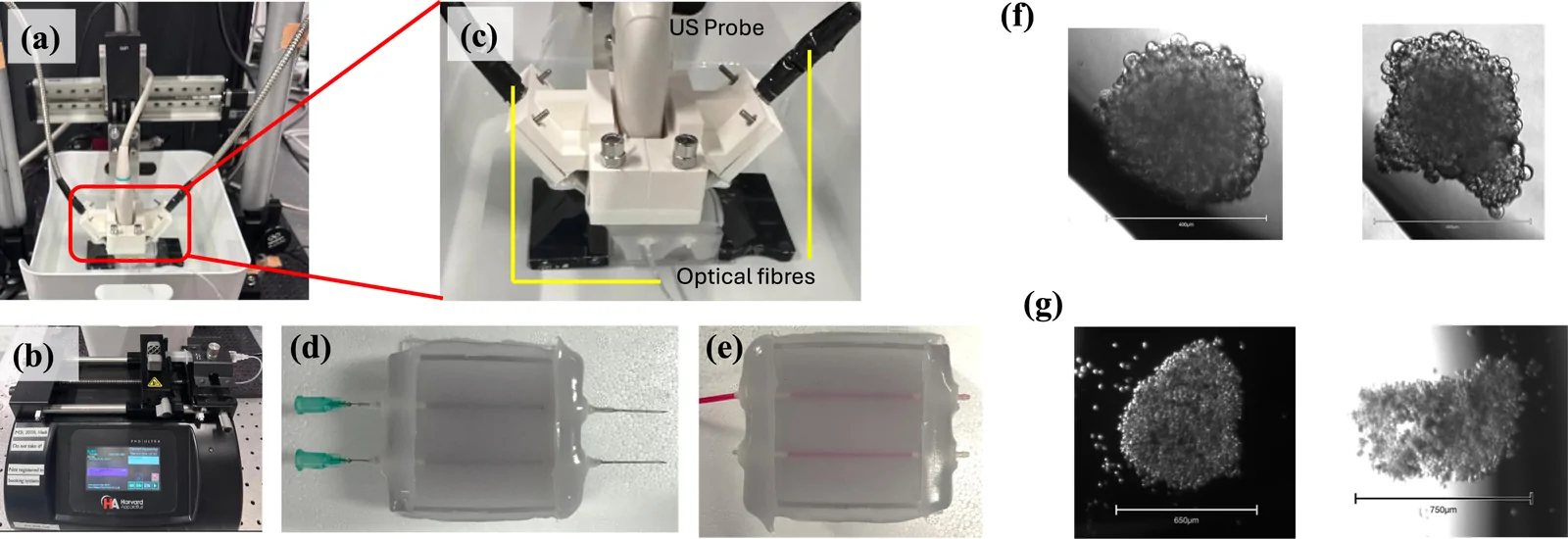
The experimental setup: (a) The stage of the photoacoustic-ultrasound imaging system; (b) the syringe pump connected to the tubes carrying donated blood or PBS; (c) the ultrasound probe and the optical fibers (linear array) placed above the lower lip phantom; (d) the lower lip phantom with channels as artery models before and (e) after removing the needles and connecting the tubes, (f) two Mel624 melanoma cell clusters; (g) two HS936.T melanoma cell clusters.

#### 2.2.2 Kymograph-based temporal analysis of CTC-like target passage

To analyze transient CTC-like target passage events in the SVD-filtered image sequences, a kymograph-based temporal analysis framework was implemented. Unlike approaches based on fixed spatial intensity sampling, the present method incorporated both temporal and spatial characteristics of the moving signal while reducing sensitivity to isolated static speckle fluctuations. The framework was developed to generate a composite temporal Target Score, $S_{\mathrm{CTC}}\left(t\right)$, with two complementary uses: (i) automated classification of complete recordings and (ii) temporal guidance of visual inspection by identifying candidate passage windows. The primary motivation was to assist the operator in reviewing long recordings by directing attention to temporal intervals in which a CTC passage was likely to occur, while the automated classification capability was additionally evaluated against the operator-identified passage status (equivalent to “ground truth” in the sensitivity-specificity analysis).

##### **Kymograph construction and Target Score**.

Foremost, a kymograph was constructed from the SVD-filtered image sequence within the manually defined vessel ROI. For each frame t and horizontal position x, the pixel intensities along the vessel cross-section were summarized using a high percentile statistic:

(1) $$K\left(x,t\right)=P_{99}\left(I_{\mathrm{ROI}}\left(x,y,t\right)\right),$$

where $P_{99}$ denotes the 99th percentile and $I_{\mathrm{ROI}}$ represents the SVD-filtered intensity values inside the ROI. This operation emphasized localized transient bright structures while reducing the influence of diffuse background fluctuations.

To suppress stationary spatial structures and enhance temporally varying features, the temporal median at each spatial position was subtracted:

(2) $$K_{\mathrm{norm}}\left(x,t\right)=K\left(x,t\right)-{\mathrm{median}}_{t}\left(K\left(x,t\right)\right).$$

The resulting kymograph was subsequently normalized using a robust median absolute deviation (MAD)-based scaling:

(3) $$K_{z}\left(x,t\right)=\frac{K_{\mathrm{norm}}\left(x,t\right)}{1.4826\;\mathrm{MAD}\;\left(K_{\mathrm{norm}}\right)+ɛ},$$

where ɛ is a small constant preventing division by zero and 1.4826 is the Gaussian consistency factor for MAD.

Spatial and temporal Gaussian smoothing were then applied using Gaussian standard deviations of 1.5 pixels in the spatial dimensions and 1.0 frame in the temporal dimension:

(4) $$K_{\mathrm{smooth}}\left(x,t\right)=G_{\sigma_{t},\sigma_{x}}*K_{z}\left(x,t\right),$$

where $\sigma_{x}=1.5$ pixels and $\sigma_{t}=1.0$ frame.

Several complementary temporal features were extracted from the processed kymograph to characterize candidate CTC passage events. Since the SVD filter suppresses the background in frames containing consistently moving objects, those frames appear dark. Therefore, firstly, a dark-band signal was computed from the negative temporal median of the kymograph:

(5) $$D\left(t\right)=-{\mathrm{median}}_{x}\left(K_{\mathrm{smooth}}\left(x,t\right)\right),$$

which quantified temporally coherent SVD-induced darkening events.

In those dark frames, moving objects such as CTCs appear as localized bright regions, ideally forming a diagonal trajectory in the kymograph. Thus, secondly, localized transient bright structures were enhanced by subtracting a slowly varying spatial background from each kymograph row:

(6) $$B\left(x,t\right)=K_{\mathrm{smooth}}\left(x,t\right)-{\mathrm{median}}_{x,\mathrm{local}}\left(K_{\mathrm{smooth}}\left(x,t\right)\right),$$

followed by rectification:

(7) $$B^{+}\left(x,t\right)=\max\left(B\left(x,t\right),0\right).$$

This operation acts as a robust spatial high-pass filter. Broad intensity variations along the vessel, slowly varying background structure, and diffuse changes in the SVD-filtered signal are suppressed, whereas compact positive residuals are retained. The rectification step ensures that only bright localized features contribute to this component, while dark bands or negative residuals are excluded.

A temporal bright-feature signal was then extracted as:

(8) $$L\left(t\right)=P_{99}\left(B^{+}\left(x,t\right)\right),$$

where the 99th percentile was used instead of the maximum to quantify strong localized bright responses while reducing sensitivity to isolated noisy pixels. Thus, L(t) increases when a compact bright object, such as a passing CTC, appears in the ROI, but remains less sensitive to global darkening or broad background fluctuations.

The direction of flow in all datasets was from right to left. To assess directional motion consistency, consecutive kymograph rows were compared under horizontal spatial shifts. The maximum tested shift was 12 pixels. Positive values of the resulting directional metric indicated preferential right-to-left motion consistency.

(9) $$M\left(t\right)=\underset{\Delta x<0}{\max}C_{\Delta x}\left(t\right)-\max\left[\underset{\Delta x>0}{\max}C_{\Delta x}\left(t\right),C_{0}\left(t\right)\right],$$

where $C_{\Delta x}\left(t\right)$ denotes the normalized correlation between consecutive kymograph rows under spatial shift Δx.

The three component signals were robustly normalized and combined into a composite Target Score. The relative contribution of each component was defined by weights $w_{\mathrm{dark}}$, $w_{\mathrm{bright}}$, and $w_{\mathrm{direction}}$:

(10) $$S_{\mathrm{CTC}}\left(t\right)=w_{\mathrm{dark}}D^{+}\left(t\right)+w_{\mathrm{bright}}L^{+}\left(t\right)+w_{\mathrm{direction}}M^{+}\left(t\right),$$

where the superscript ${\left(\cdot \right)}^{+}$ denotes rectification after robust normalization. The final weights were $w_{\mathrm{dark}}=0.60$, $w_{\mathrm{bright}}=0.15$, and $w_{\mathrm{direction}}=0.25$. The final weights were $w_{\mathrm{dark}}=0.60$, $w_{\mathrm{bright}}=0.15$, and $w_{\mathrm{direction}}=0.25$. The weights and the final Target Score threshold were evaluated through a systematic parameter sweep using the operator-identified passage status as the reference classification. The Dark, Bright, and Direction weights were varied in increments of 0.05, subject to the constraint that their sum was equal to one, while the Target Score threshold was varied from 0.5 to 5.5 in increments of 0.25. The selected parameter combination achieved the maximum observed F1 score in this dataset and was retained for the primary analysis. Given the limited number of experimental cases, this analysis was intended to assess parameter sensitivity rather than provide independent validation of generalization performance. The complete weight and threshold sensitivity analysis is provided in **Supplementary Materials B**. The final threshold for automated classification was $S_{\mathrm{CTC}}=4.0$.

The composite Target Score was subsequently used in two ways. For automated classification, candidate events were identified from the continuous score and compared with the final threshold to assign each recording as positive or negative. For temporal guidance, the same score trace was used to identify candidate temporal intervals for focused visual inspection, including intervals that remained below the automatic classification threshold.

##### **(i) Automated classification**.

For automated classification, candidate temporal events were first identified independently of the final Target Score weights, using the strongest response among the three component signals. A minimum peak prominence of 1.0 and a minimum peak separation of 0.40 s were applied to identify distinct candidate peaks. For each detected peak, an event envelope was then defined to determine the temporal extent of the corresponding candidate event. The envelope extended on either side of the peak for as long as the signal remained above a local amplitude threshold defined as the greater of 45% of the peak amplitude and an absolute floor of 1.20. This procedure allowed the duration of each event to adapt to its peak magnitude while preventing very small peaks from producing excessively broad or unstable envelopes.

For each candidate event, the maximum composite Target Score within the event envelope was compared with the final threshold of $S_{\mathrm{CTC}}=4.0$. Events reaching or exceeding this threshold were classified as automatically detected. For experiment-level evaluation, the reference endpoint was defined by operator-identified passage status rather than by biological target presence or absence. An experiment was classified as *operator-positive (OP)* when the operator visually identified a CTC passage, and as *operator-negative (ON)* when no visually identifiable passage was observed. The algorithm output was classified as *algorithm-positive (AP)* or *algorithm-negative (AN)* according to whether at least one candidate event reached or exceeded the final Target Score threshold, or no such event was identified, respectively. Importantly, an operator-negative condition indicates only that no visually identifiable passage was observed and does not imply absence of experimentally introduced cells or clusters.

The four possible combinations of operator reference and algorithm output were denoted OP–AP, OP–AN, ON–AP, and ON–AN. These correspond, respectively, to agreement on an operator-identified passage, a missed operator-identified passage, an algorithm-positive classification without an operator-identified passage, and agreement on the absence of an operator-identified passage. For calculation of sensitivity, specificity, precision, and F1 score, these four categories correspond mathematically to true-positive (TP), false-negative (FN), false-positive (FP), and true-negative (TN) outcomes, respectively, with the operator-identified passage status serving as the binary reference. Thus, the resulting metrics quantify agreement with the operator reference and should not be interpreted as measures of sensitivity or specificity for the biological presence of CTCs.

##### **(ii) Temporal guidance for visual inspection**.

The second use of the composite Target Score was to guide visual inspection of the complete recorded movies. The complete movies were retained for visual assessment, while the temporal score $S_{\mathrm{CTC}}\left(t\right)$ provided an additional guide by highlighting time intervals containing candidate passage events for closer examination. This is particularly useful for long recordings, for which reviewing the entire movie frame-by-frame can make brief transient events difficult to identify. The corresponding kymograph provides complementary spatial–temporal information, allowing the inspector to assess the temporal evolution and spatial coherence of highlighted events.

For visual guidance, candidate temporal windows with a duration of at least 0.5 s were highlighted in the $S_{\mathrm{CTC}}\left(t\right)$ trace. This duration was selected to approximately match the expected transit time through the imaged spatial region at the experimental flow speed. Importantly, this duration criterion was applied only to the displayed guidance windows and did not modify the underlying candidate-event detection or the automatic classification.

For each displayed candidate, the maximum Target Score within its temporal interval was compared with the final automatic detection threshold of $S_{\mathrm{CTC}}=4.0$. Candidate windows for which the maximum $S_{\mathrm{CTC}}$ within the temporal interval was below 4.0 were displayed in light blue, whereas windows reaching $S_{\mathrm{CTC}}\geq 4.0$ were displayed in dark blue. Thus, sub-threshold candidate events were retained as visual prompts rather than discarded, while the dark-blue regions corresponded to events that also satisfied the automatic detection criterion. Together with the kymograph, these highlighted intervals provided a focused temporal and spatial guide for closer examination of the complete recordings.

For quantitative evaluation of the temporal guidance, overlapping candidate windows were merged before counting the number of flags. Thus, multiple overlapping candidate intervals associated with the same temporal region were treated as a single guidance flag. A merged candidate window was considered a true temporal-guidance flag if it overlapped an operator-identified CTC passage; otherwise, it was considered a false flag. Experiments for which no temporal CTC passage was identified by the operator were not assigned a ground-truth passage window, and all candidate flags in these recordings were therefore considered false guidance flags.

The operator performing the visual inspection first independently marked the temporal windows corresponding to all visually identified CTC passages using a MATLAB-based helper tool, which allowed selection of the start and end frames of each passage directly from the recorded image sequence. These operator-defined passage windows were subsequently used as the temporal operator-identified passage status for evaluation of the guidance performance. The effectiveness of temporal guidance was quantified using passage hit rate, passage coverage, and temporal intersection-over-union (IoU). The passage hit rate was defined as

(11) $$H_{\mathrm{passage}}=\frac{N_{\mathrm{hit}}}{N_{\mathrm{GT}}},$$

where $N_{\mathrm{hit}}$ is the number of operator-identified CTC passages for which at least one merged candidate window overlapped the passage, and $N_{\mathrm{GT}}$ is the total number of operator-identified CTC passages with valid temporal boundaries.

For each operator-identified passage, passage coverage was calculated from the temporal overlap between the best-matching merged candidate window and the operator-defined passage:

(12) $$C_{\mathrm{passage}}=\frac{T_{\mathrm{overlap}}}{T_{\mathrm{GT}}},$$

where $T_{\mathrm{overlap}}$ is the duration of the temporal intersection between the candidate and ground-truth windows, and $T_{\mathrm{GT}}$ is the duration of the operator-defined passage. Passage coverage therefore quantifies how much of the visually identified passage was included in the temporal guidance window.

Temporal localization was additionally quantified using the intersection-over-union (IoU):

(13) $$\mathrm{IoU}=\frac{T_{\mathrm{overlap}}}{T_{\mathrm{candidate}}+T_{\mathrm{GT}}-T_{\mathrm{overlap}}},$$

where $T_{\mathrm{candidate}}$ is the duration of the best-matching merged candidate window. In contrast to passage coverage, IoU penalizes candidate windows that extend substantially beyond the operator-defined passage. Thus, a candidate window can achieve high passage coverage while having a lower IoU if it successfully contains the complete passage but covers a substantially broader temporal interval.

The mean passage coverage was calculated across all operator-identified passages, whereas the median IoU was reported to reduce the influence of individual broad candidate windows.

Finally, the reduction in temporal search space provided by the guidance was quantified by calculating the fraction of each complete recording covered by the union of the merged candidate windows. The targeted inspection fraction was defined as

(14) $$F_{\mathrm{search}}=\frac{T_{\mathrm{flagged}}}{T_{\mathrm{video}}},$$

where $T_{\mathrm{flagged}}$ is the total duration of the non-overlapping merged candidate windows and $T_{\mathrm{video}}$ is the complete recorded duration. The corresponding temporal search-space reduction was defined as

(15) $$R_{\mathrm{search}}=1-F_{\mathrm{search}}.$$

The search-space metrics were calculated for each experiment and also pooled across all recordings using the summed flagged and recorded durations. This metric quantifies the practical benefit of the temporal score for long recordings: a lower targeted inspection fraction corresponds to a greater reduction in the amount of video requiring detailed frame-by-frame examination, while passage hit rate and temporal overlap metrics quantify whether the reduction in inspected time is achieved without overlooking visually identifiable passage events.

### 2.3 Phantom development

A tissue-mimicking phantom was developed to replicate some of the important anatomical and physical properties of the inferior labial region. The vascular structure consisted of a bare cylindrical channel molded directly into the copolymer matrix; no embedded tube was used. The channel diameter was 1 mm and its center was positioned approximately 1–2 mm below the inner labial surface. These dimensions were selected to provide a practical approximation of the shallow inferior labial vasculature while allowing reliable optical and acoustic access. Ultrasonographic studies of the inferior labial artery demonstrate that the vessel is located superficially within the lower lip and provide anatomical measurements supporting the use of a shallow vascular model [22], [23].

#### 2.3.1 Optical and acoustic properties

The optical properties of the phantom were selected to approximate mucosal tissue, based on reported optical properties of human stomach mucosa and human colon tissue [24], [25]. A copolymer-in-oil formulation was prepared following an established approach for photoacoustic tissue-mimicking phantoms [26], consisting of low-density polyethylene (LDPE, Thermo Fisher Scientific, USA), polystyrene-block-poly(ethylene-ran-butylene)-block-polystyrene (SEBS, Sigma-Aldrich, USA), and mineral oil (Sigma-Aldrich, USA) in weight fractions of 8%, 15%, and 77%, respectively. Titanium dioxide (TiO_2_, anatase, Sigma-Aldrich, USA) and nigrosin (Sigma-Aldrich, USA) were incorporated as scattering and absorbing agents at concentrations of 0.16% (w/v) and 0.0022% (w/v), respectively, yielding target optical properties at 700 nm of an absorption coefficient ($\mu_{a}$) of approximately 0.005 mm^−1^ and a reduced scattering coefficient ($\mu_{s}^{\prime }$) of approximately 1.3 mm^−1^, in agreement with properties of mucosal tissue. The resulting phantom exhibited a speed of sound of approximately 1480 m/s, corresponding to an ∼8% deviation from the reported value of 1520 m/s for mucosal tissue while maintaining fabrication stability and reproducibility [26]. To ensure homogeneity and reduce optical artifacts from trapped air, the phantom was degassed in a vacuum oven prior to use.

#### 2.3.2 Flow setup

Flow within the phantom channel was established using a syringe pump (Harvard Apparatus, USA) to mimic physiological blood flow conditions. The flow velocity was set to approximately 30 mm/s, selected to represent flow conditions relevant to the shallow vessels of the lower lip. Reported labial vascular flow measurements span approximately 8–50 mm/s, with values around 30 mm/s providing a representative condition for the present feasibility experiments [27].

To ensure adequate temporal sampling of moving targets, the laser pulse repetition rate (PRR) must satisfy:

(16) $$f\geq \frac{V}{D+d},$$

where V is the flow velocity, D is the characteristic size of the target, and d is the width of the optical excitation beam [28]. For the experimental flow velocity of 30 mm/s and an effective optical excitation beam width of approximately 20 mm, Eq. (16) gives a minimum PRR of approximately 1.5 Hz for a 20-μm target and 1.4–1.5 Hz for the 0.5–1 mm clusters investigated here. The 100 Hz PRR therefore provides substantially more frequent temporal sampling than required by this criterion. This calculation was used to demonstrate adequate temporal sampling under the experimental conditions and does not establish a general detection limit across all possible target velocities and sizes.

Cell suspensions, microspheres, and blood samples were injected into the system through connected tubing. Prior to each experiment, the system was flushed with water and air to minimize residual contamination and ensure reproducibility (see Table 2).

**Table 2:** Summary of the tissue-mimicking phantom geometry, optical and acoustic properties, flow conditions, and blood preparation used for photoacoustic detection experiments.

Parameter	Value/Description
Channel geometry	Bare cylindrical channel molded directly into the phantom
Channel diameter	1 mm
Channel depth	Approximately 1–2 mm below the inner labial surface
Base phantom composition	8 wt% LDPE, 15 wt% SEBS, 77 wt% mineral oil
Scattering agent	TiO_2_ (anatase)
Absorbing agent	Nigrosin
Absorption coefficient (700 nm)	$\mu_{a}\approx 0.005\;{\mathrm{mm}}^{-1}$
Reduced scattering coefficient (700 nm)	$\mu_{s}^{\prime }\approx 1.3\;{\mathrm{mm}}^{-1}$
Speed of sound	c≈ 1480 m/s
Degassing procedure	Vacuum oven degassing prior to use
Flow generation	Syringe pump (Harvard Apparatus, USA)
Flow velocity	V≈ 30 mm/s
Blood preparation	Human donor blood collected in $K_{2}$-EDTA tubes

#### 2.3.3 Control microspheres

Black polymer microspheres (Cospheric, BKPMS-1-2; 75–90μm diameter; density 1.2 g/cm^3^) were used as an initial non-biological absorber control. These microspheres were selected because their strong optical absorption and size below that of the smallest biological clusters investigated (100μm) provided a conservative test of the photoacoustic system’s ability to capture and track small, moving absorbing particles before proceeding to the more challenging biological experiments.

#### 2.3.4 Donated human blood

To evaluate detection performance under physiologically relevant conditions, experiments were conducted using blood samples obtained from healthy volunteers recruited at the University of Twente under institutional protocols facilitated by the TechMed Centre. Ethical approval for the use of donated human blood was obtained from the Ethics Committee Natural Sciences and Engineering Sciences (NES) at the University of Twente. Blood samples were collected in $K_{2}$-EDTA anticoagulant tubes (purple-top vacutainers; BD) to prevent coagulation. For phantom experiments, blood was introduced into the channel either alone or spiked with melanoma cells or multicellular clusters at the concentrations specified for each experimental condition. For the single-cell experiment, $1\times 10^{6}$ melanoma cells were suspended in 15 mL of blood, corresponding to approximately $6.7\times 10^{4}$ cells/mL. For the multicellular-cluster experiments, blood was spiked with 40 multicellular clusters per mL. The presence of blood provided a realistic optical background dominated by hemoglobin absorption, representing a major challenge for photoacoustic detection of melanoma cells.

Because access to donated human blood and the available number of melanoma cells was limited, the experiments were designed as a feasibility study rather than as a large-scale statistical characterization of the detection limit.

### 2.4 Cell culture and cluster preparation

Human melanoma cell lines, Mel624 and HS936.T, were kindly provided by A. de Boer (Radboud University, Nijmegen, The Netherlands) and Dr. John Viator (Duquesne University), respectively. Cells were cultured in Dulbecco’s Modified Eagle Medium (DMEM; Gibco) with 5% fetal bovine serum (FBS; Gibco) and penicillin (10 U/mL)/streptomycin (10μg/mL). Mel624 cells were treated for 96 h with 100 nM α-melanocyte-stimulating hormone (α-MSH, Merck 05-23-0751) to increase melanin production [29]. The cell-culture protocol used a component derived from bovine fetuses. Alternative culture approaches using human-derived supplements, including human serum [30], are being investigated and could help avoid reliance on animal-derived materials in future studies, thereby providing a more ethically preferable approach to cell culture.

To compare relative melanin content between the melanoma cell conditions, $1\times 10^{6}$ cells of HS936.T, untreated Mel624, or Mel624 treated with 100 nM α-MSH were seeded in 12-well plates and cultured in triplicate. Following incubation, the culture medium was removed and cells were lysed by adding 1 N NaOH containing 10% (v/v) DMSO. The lysates were transferred to microcentrifuge tubes and incubated for 2 h at 80°C to solubilize melanin [31]. After incubation, samples were cooled to room temperature and the optical absorbance spectrum (200–1000 nm) of each lysate was measured using a plate reader (SpectraMax iD3). These measurements represent relative optical absorbance rather than an absolute melanin concentration. The spectra were used to establish the relative difference in melanin content between the cell conditions, with particular relevance to the 700 nm excitation wavelength used for photoacoustic imaging.

For each sample condition, the optical absorbance spectrum was measured in triplicate at each of the 78 investigated wavelengths. Values are presented as mean ± standard error of the mean (SEM), calculated from the three (n=3) measurements at each wavelength. Pairwise comparisons at 700 nm were performed using two-tailed t-tests in Excel. The reported p-values were not corrected for multiple comparisons.

For phantom experiments, cells were prepared either as single-cell suspensions or as multicellular clusters. Cell clusters were generated using the hanging-drop method by seeding 1000–2500 cells in 25μL drops and incubating for 96 h, resulting in clusters of varying sizes [32]. Prior to injection into the phantom, both single cells and clusters were resuspended in phosphate-buffered saline (PBS, pH 7.4; Gibco). The presence of melanin provides intrinsic optical absorption contrast, enabling detection using photoacoustic imaging [21].

For the cluster experiments, targets included single melanoma cells and multicellular clusters with characteristic diameters of approximately 500, 700, and 1000μm. The 500μm HS936.T cluster condition was additionally evaluated in blood. The limited availability of donated blood and cells prevented a systematic evaluation across a larger range of cluster sizes and melanin contents, and each experimental condition was therefore measured only once.

## 3 Results

### 3.1 Photoacoustic detection of melanoma cell targets

Representative photoacoustic images of the investigated melanoma-cell targets, together with the corresponding SVD-filtered image sequences, are shown in Fig. 3a. The optical absorbance measurements showed significantly higher signals for both HS936.T and Mel624+α-MSH cells compared with the control medium across the investigated wavelength range (Fig. 3b–c). The spectra are presented as mean ± SEM, where the error bars represent the standard error of the mean calculated from the three independent measurements at each wavelength. At the investigated wavelength of 700 nm, both cell types showed significantly higher melanin-related optical absorption than the control, with two-tailed t-test p-values of 0.0025 and 0.0045 for HS936.T and Mel624+α-MSH, respectively. The difference between the two melanoma-cell types was also statistically significant (p=0.0065).

The SVD-based processing workflow is illustrated in Fig. 3d, showing the original photoacoustic frame, the manually selected vessel/lumen ROI, and the resulting SVD-filtered frame. Following SVD filtering and extraction of the Dark, Bright, and Direction components, the composite Target Score $S_{\mathrm{CTC}}\left(t\right)$ was evaluated across the complete set of melanoma-cell experiments. The visual detectability and algorithmic classification results are summarized in Table 3.

Single HS936.T cells (20μm) were not visually detected in PBS. For HS936.T clusters in PBS, clusters of 1000μm, 700μm, and 500μm were visually detected, whereas the 100μm cluster was not. The 500μm HS936.T cluster in blood was also not visually detected. Mel624+α-MSH clusters of 500μm were visually detected at 500μm in both PBS and blood, whereas the 100μm clusters were not detected in either medium. The Mel624 cluster without α-MSH at 500μm in PBS was visually detected.

**Fig. 3:**
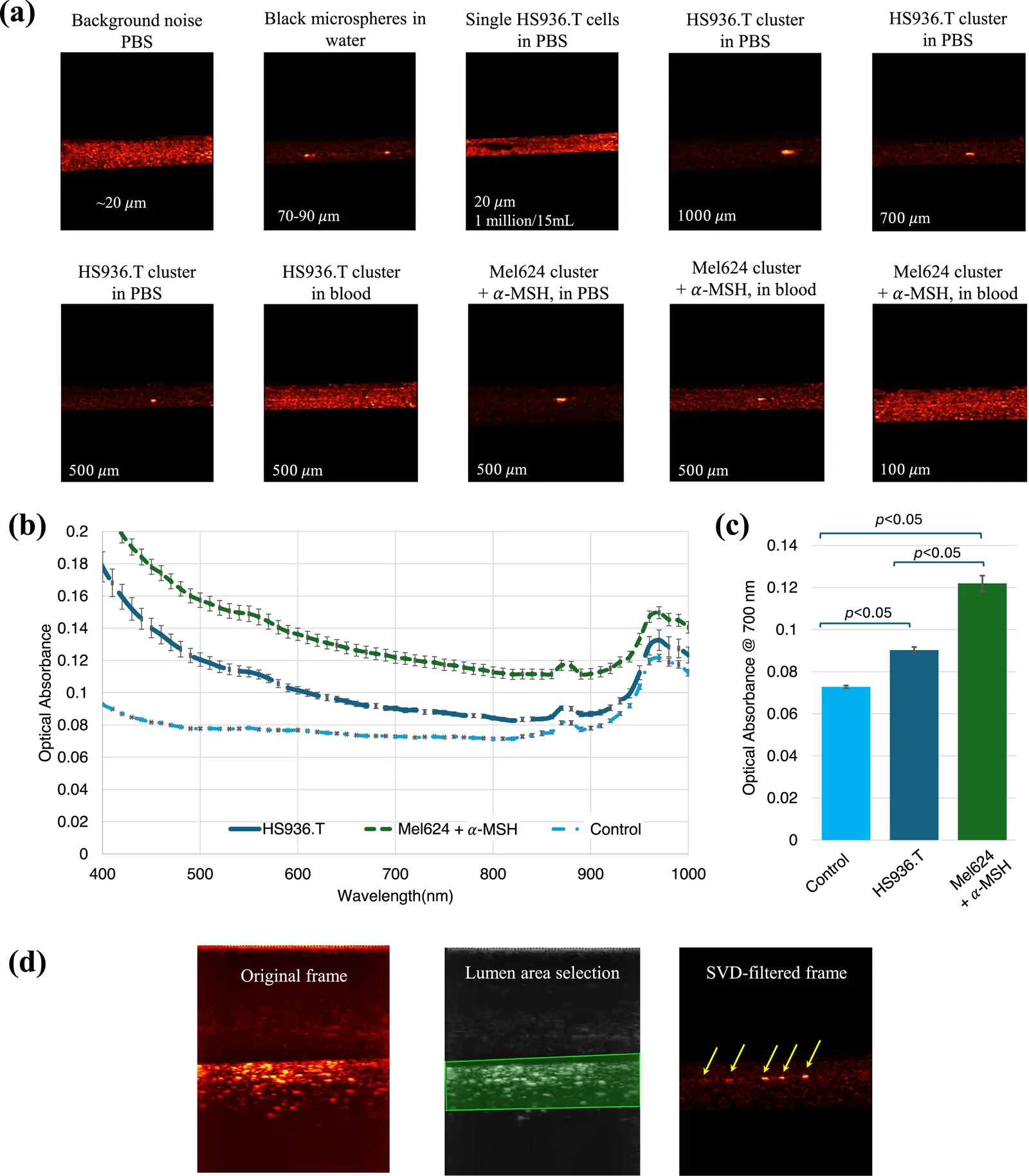
Photoacoustic visualization and melanin-content characterization of melanoma-cell targets investigated in the lower-lip phantom experiments. (a) Representative examples of the investigated melanoma-cell targets and corresponding SVD-filtered photoacoustic image sequences during target passage through the vessel-mimicking channels. (b) Optical absorbance spectra of HS936.T and Mel624+α-MSH melanoma cells compared with the control medium across the wavelength range from 400 to 1000 nm. Error bars represent the SEM calculated from three independent measurements at each wavelength. (c) Bar-plot representation of the optical absorbance of the investigated cell types and control medium at 700 nm, used as an indicator of relative melanin content. The p-values are based on two-tailed t-tests and are as follows: $p_{Control-HS936.T}=0.0025$, $p_{Control-Mel624+\alpha -MSH}=0.0045$, and $p_{HS936.T-Mel624+\alpha -MSH}=0.0065$. (d) Example of the SVD-based processing workflow for black microspheres in water, showing the original photoacoustic frame, the manually selected vessel/lumen ROI, and the corresponding SVD-filtered frame.

Using the final decision threshold of $S_{\mathrm{CTC}}=4.0$, the algorithm agreed with the operator-identified passage status in nine of the eleven experimental conditions, with four operator-positive conditions classified as algorithm-positive and five operator-negative conditions classified as algorithm-negative. Two operator-positive conditions were classified as algorithm-negative, and no operator-negative conditions were classified as algorithm-positive. This resulted in four OP–AP, five ON–AN, two OP–AN, and no ON–AP outcomes, corresponding to an operator-referenced sensitivity of 0.667, specificity of 1.000, precision of 1.000, accuracy of 0.818, and F1 score of 0.800. The receiver operating characteristic analysis yielded an operator-referenced AUC of 0.733.

**Table 3:** Operator-referenced classification of the experimentally introduced melanoma cells and clusters. OP, operator-positive (a visually identifiable passage was observed); ON, operator-negative (no visually identifiable passage was observed); AP, algorithm-positive; AN, algorithm-negative. The outcome labels describe the relationship between the operator reference and algorithm classification.

Target	Size	Medium	Operator reference	Algorithm	Outcome
Single HS936.T cell	20μm	PBS	ON	AN	ON–AN
HS936.T cluster	1000μm	PBS	OP	AP	OP–AP
HS936.T cluster	700μm	PBS	OP	AP	OP–AP
HS936.T cluster	500μm	PBS	OP	AN	OP–AN
HS936.T cluster	500μm	Blood	ON	AN	ON–AN
HS936.T cluster	100μm	PBS	ON	AN	ON–AN
Mel624 cluster + α-MSH	500μm	PBS	OP	AP	OP–AP
Mel624 cluster + α-MSH	100μm	PBS	ON	AN	ON–AN
Mel624 cluster + α-MSH	500μm	Blood	OP	AN	OP–AN
Mel624 cluster + α-MSH	100μm	Blood	ON	AN	ON–AN
Mel624 cluster	500μm	PBS	OP	AP	OP–AP

**Overall classification: OP–AP = 4, ON–AN = 5, ON–AP = 0, OP–AN = 2**					
**Operator-referenced sensitivity = 0.667, specificity = 1.000, precision = 1.000, accuracy = 0.818, F1 = 0.800**					
**Operator-referenced AUC = 0.733**					

The dependence of F1 score and precision on the decision threshold is shown in Fig. 5b, where the F1 score and precision are plotted as functions of $S_{\mathrm{CTC}}$. The final threshold of 4.0 was used for the binary classifications reported in Table 3.

### 3.2 Kymograph-based Target Score analysis

Representative spatial–temporal examples of the Target Score are shown in Fig. 4. In the Mel624+α-MSH 500μm blood experiment (Fig. 4b), a dark band and coherent diagonal trajectory are visible in the kymograph, accompanied by a pronounced peak in $S_{\mathrm{CTC}}\left(t\right)$. The peak, however, remains below the final threshold of 4.0, and the experiment is therefore classified as OP-AN. The HS936.T 500μm blood experiment (Fig. 4c) shows weaker and more fragmented fluctuations without a clear coherent trajectory, with the corresponding score remaining below the threshold and resulting in a ON-AN classification. In contrast, the Mel624+α-MSH 500μm PBS experiment (Fig. 4d) shows a pronounced dark band and coherent trajectory accompanied by a score peak above 4.0, resulting in a OP-AP classification. Note that in the kymograph plots, the width of a dark or bright band reflects the spatial extent of the corresponding signal, while the slope of a diagonal trajectory reflects the passage velocity of the moving target. With a less speckled background, the dark band and the trajectory appear less noisy with clearer margins.

The Target Score traces also show candidate temporal intervals below the automatic classification threshold. Light-blue regions identify candidate intervals that remain below $S_{\mathrm{CTC}}=4.0$, whereas dark-blue regions identify candidate intervals reaching the automatic detection threshold. These candidate intervals could be used for the visual inspection by the operator to double check the flagged windows in a scenario where the ground truth is not known to the operator.

**Fig. 4:**
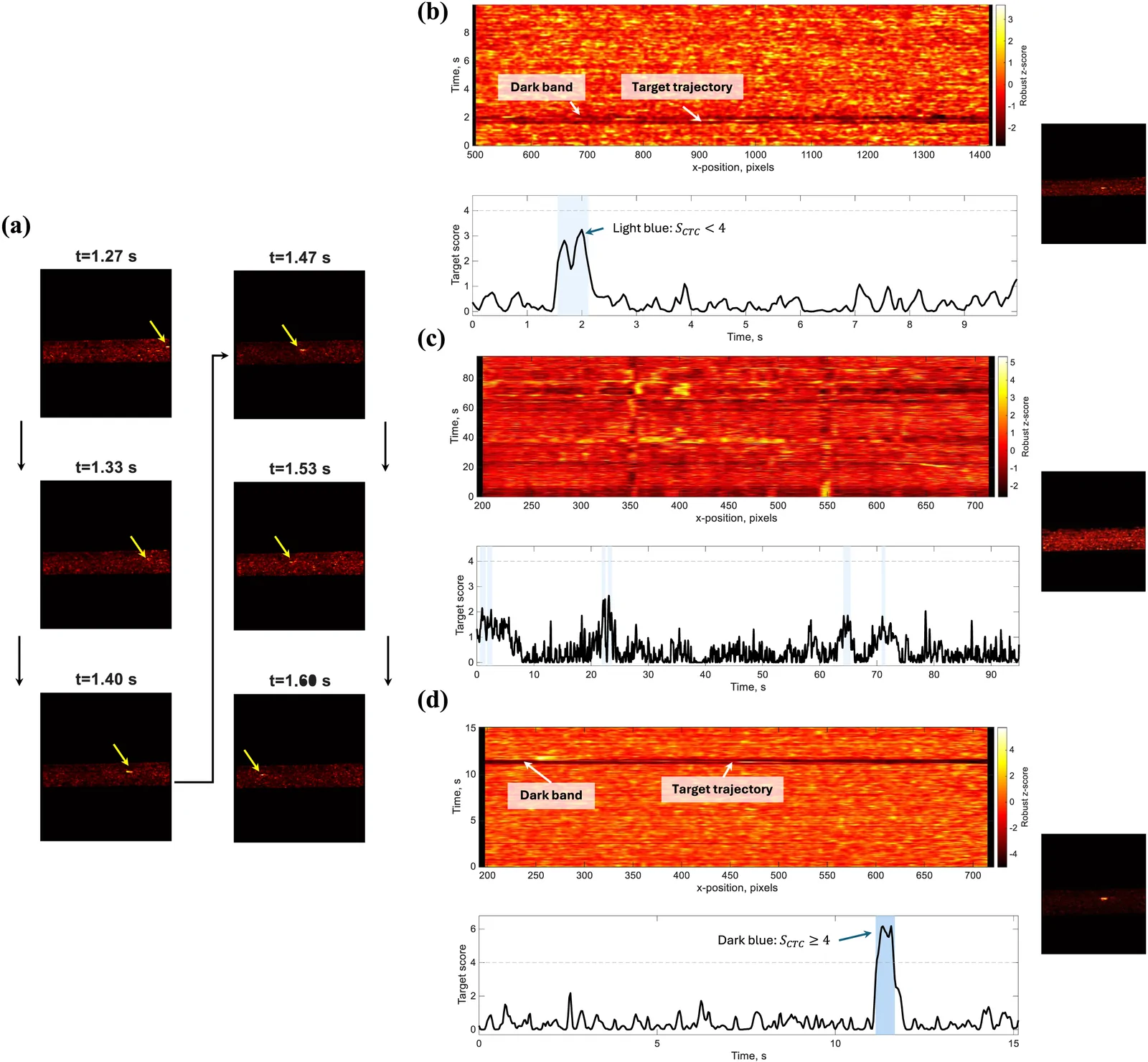
Kymograph-based temporal analysis and Target Score assessment of transient melanoma-cluster passage events. (a) Consecutive SVD-filtered photoacoustic frames showing passage of a Mel624+α-MSH cluster (500μm) in blood through the vessel-mimicking channel. (b–d) Representative kymographs and corresponding Target Score traces for Mel624+α-MSH in blood (b), HS936.T in blood (c), and Mel624+α-MSH in PBS (d). A clear dark band and coherent trajectory are observed in (b) and (d), whereas (c) shows weaker and fragmented fluctuations. With a final Target Score threshold of $S_{\mathrm{CTC}}=4.0$, cases (b), (c), and (d) are classified as operator-positive-algorithm-negative negative (OP-AN), operator-negative-algorithm-negative (ON-AN), and operator-positive-algorithm-positive (OP-AP), respectively. Shaded regions indicate candidate temporal windows for inspection, with a minimum duration of 0.5 s imposed based on the flow speed and spatial width of the imaging window. Light-blue regions denote candidate windows below the automatic detection threshold, retained as guidance for operator inspection, whereas darker-blue regions denote candidate windows in which $S_{\mathrm{CTC}}\geq 4.0$.

### 3.3 Temporal guidance for visual inspection

The temporal guidance performance across the experiments is summarized in Table 4. Among the six experiments for which the operator identified a CTC passage with defined temporal boundaries, five passages were captured by at least one guidance window, corresponding to a passage hit rate ($H_{\mathrm{passage}}$) of 83.3%.

For the five captured passages, mean passage coverage ($C_{\mathrm{passage}}$ ) was 92.3%, while the median temporal IoU (Eq. (13)) was 0.889. The individual passage coverage values ranged from 88.9% to 100%, with IoU values ranging from 0.152 to 1.000.

Candidate windows were also generated in recordings for which no operator-defined CTC passage was available. These windows were retained as temporal guidance candidates and were counted as false guidance flags when assessing overlap with the visual operator-identified passage status. The number and status of the guidance flags for each experiment are reported in Table 4.

Across all recordings, the merged candidate windows covered 8.21% of the total recorded duration, corresponding to a search-space reduction ($R_{\mathrm{search}}$) of 91.79%.

**Table 4:** Temporal guidance performance of the composite Target Score during visual inspection. A true flag corresponds to a Target Score flag occurring within an operator-identified passage, whereas a false flag occurs outside an operator-identified passage.

Target	Size	Medium	Temporal guidance	$C_{\mathrm{passage}}$	IoU
Single HS936.T cell	20μm	PBS	1 flag (0 true, 1 false)	–	–
HS936.T cluster	1000μm	PBS	1 flag (1 true, 0 false)	100%	1.000
HS936.T cluster	700μm	PBS	7 flags (1 true, 6 false)	88.9%	0.889
HS936.T cluster	500μm	PBS	0 flags	0%	0.000
HS936.T cluster	500μm	Blood	6 flags (0 true, 6 false)	–	–
HS936.T cluster	100μm	PBS	2 flags (0 true, 2 false)	–	–
Mel624 cluster + α-MSH	500μm	PBS	1 flag (1 true, 0 false)	100%	1.000
Mel624 cluster + α-MSH	100μm	PBS	0 flags	–	–
Mel624 cluster + α-MSH	500μm	Blood	1 flag (1 true, 0 false)	92.9%	0.867
Mel624 cluster + α-MSH	100μm	Blood	3 flags (0 true, 3 false)	–	–
Mel624 cluster	500μm	PBS	2 flags (1 true, 1 false)	100%	0.152

**Passage hit rate (**$H_{\mathrm{passage}}$**) = 83.3% (5/6 passages)**					
**Mean passage coverage (**$C_{\mathrm{passage}}$**) = 92.3%, Median temporal IoU = 0.889**					
**Targeted inspection fraction (**$F_{\mathrm{search}}$**) = 8.21%, Search-space reduction (**$R_{\mathrm{search}}$**) = 91.79%**					

**Fig. 5:**
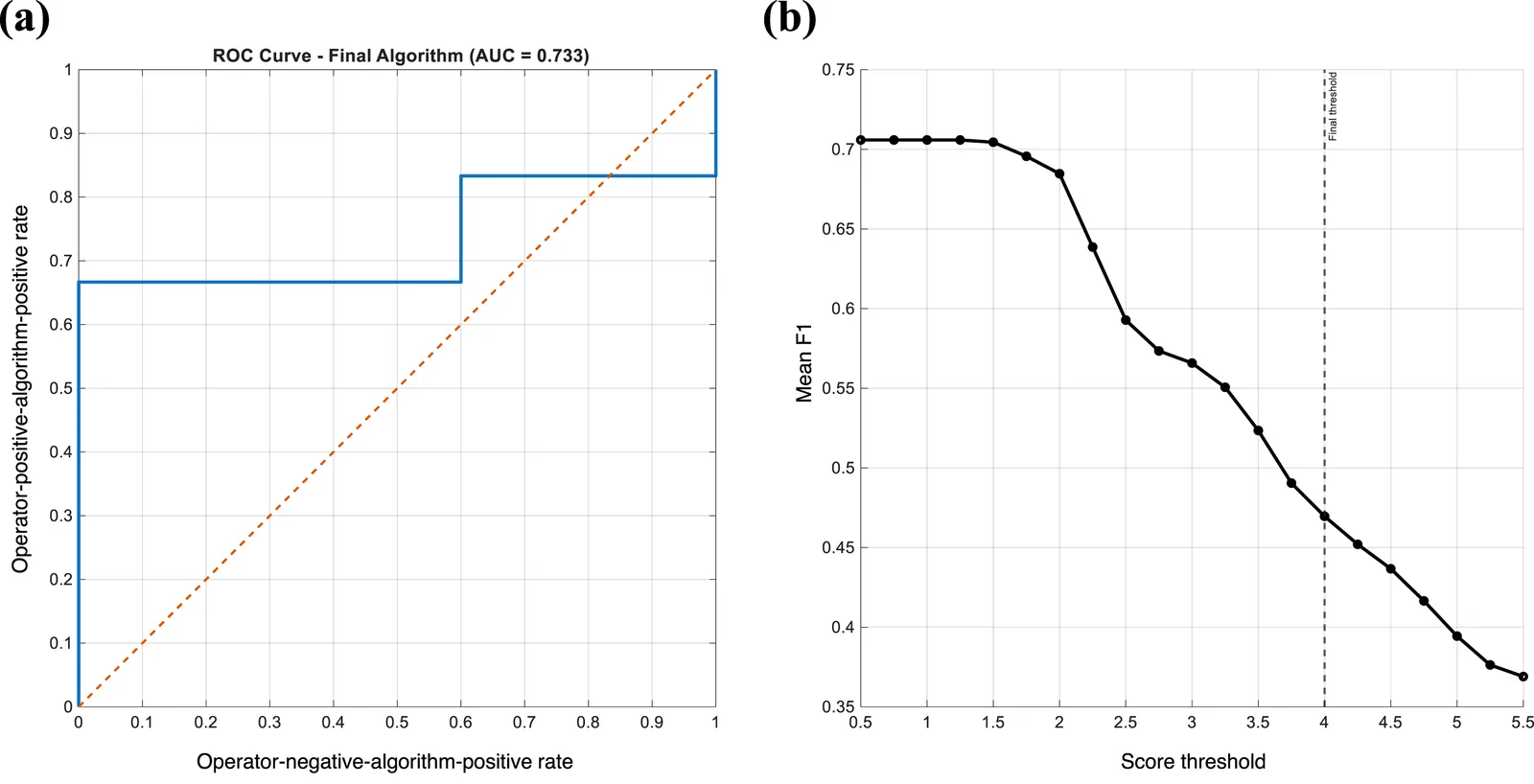
Operator-referenced classification performance of the composite Target Score. (a) Receiver operating characteristic (ROC) curve showing the operator-referenced true-positive rate versus false-positive rate across score thresholds, with the corresponding operator-referenced area under the curve (AUC). (b) Mean F1 score as a function of the $S_{\mathrm{CTC}}$ decision threshold, with the final threshold indicated.

## 4 Discussion

### 4.1 Photoacoustic detectability of melanoma-cell targets

The present study demonstrated proof-of-concept photoacoustic visualization and temporal characterization of melanoma-cell-like targets flowing through vessel-mimicking channels embedded within a lower-lip phantom. Using SVD-enhanced photoacoustic imaging together with kymograph-based temporal analysis, transient target-passage events could be visualized and temporally localized under both PBS and blood-containing conditions. The experimental configuration combined tissue-mimicking geometry and material, dynamic flow conditions, donated human blood, and cultured melanoma-cell targets, thereby providing a more physiologically relevant environment compared with static phantom measurements.

The observed detectability depended on target size, pigmentation, and surrounding medium. Larger HS936.T clusters were visually detectable in PBS, whereas the single 20μm cell and the 100μm cluster were not. The 500μm HS936.T cluster was also not visually detected in blood. In comparison, the 500μm Mel624+α-MSH cluster was detectable in both PBS and blood, while the corresponding 100μm cluster was not. These observations indicate that both target size and optical absorption characteristics influence the visibility of transient melanoma-cell-like targets under the investigated conditions.

The size dependence is consistent with the larger absorbing volume of cell clusters producing stronger photoacoustic responses. However, the results also suggest that target size alone does not determine detectability. Mel624+α-MSH exhibited higher melanin content than HS936.T across the investigated wavelength range, as shown in Fig. 3, which provides a plausible explanation for the stronger detectability of the pigmented Mel624+α-MSH clusters. The enhanced optical absorption associated with higher melanin content can increase the photoacoustic response and thereby improve separation from the surrounding background.

In the present study, α-MSH stimulation was used to enhance melanogenesis through a physiologically relevant melanocortin signaling pathway associated with melanin production in melanocytic and melanoma-cell models [33], [34]. Previous studies have shown that α-MSH binding to MC1R activates cAMP/MITF-mediated melanogenesis pathways and increases melanin synthesis in melanocytes and melanoma-related cell models [33], [34]. The resulting increase in pigmentation provides a useful experimental means of investigating the influence of melanin-associated optical absorption on photoacoustic detectability. However, the present experiments do not establish a quantitative relationship between melanin content and photoacoustic detectability because of the limited number of experimental cases.

The reduced detectability observed in blood compared with PBS further highlights the challenge posed by the background environment. Blood introduces additional optical absorption and temporally varying photoacoustic background signals, which can reduce the contrast between a transient target and its surroundings. This effect was particularly evident for the less pigmented HS936.T cluster in blood. Conversely, the detection of the highly pigmented Mel624+α-MSH cluster in blood suggests that increased optical absorption can partially compensate for the more challenging background.

### 4.2 Contribution of SVD filtering and kymograph-based Target Score analysis

#### 4.2.1 SVD filtering

SVD-based background suppression played an important enabling role in the visualization of transient target-passage events. Following SVD filtering, stationary background components were reduced and transient moving features became more visually distinguishable within the vessel ROI, as shown in Fig. 3-(d). The additional quantitative analysis in **Supplementary Materials A** provides independent support for this observation. Across the six visually identified passage events (cases with the tag Yes under Visual Inspection in Table 3), SVD filtering increased both SBR (signal-to-background ratio) and CNR (contrast-to-noise ratio) in all six paired experiments. The median case-level SBR increased from 1.358 without SVD to 2.701 with SVD, while the median CNR increased from 0.351 to 2.088. The paired differences were significant by two-sided Wilcoxon signed-rank testing (p=0.03125 for both metrics). These results indicate that SVD filtering consistently improved quantitative signal–background separation under the investigated conditions. Detailed calculations and the percentile sensitivity analysis can be found in **Supplementary Materials A**.

The improvement remained consistent when the percentile used to define the temporally varying signal region was varied from the 90th to the 99th percentile. This suggests that the observed advantage of SVD filtering was not dependent on the specific 95th-percentile setting used for the primary analysis. Nevertheless, these results should be interpreted as evidence for improved signal–background separation rather than as direct evidence of improved clinical CTC detection, because the quantitative analysis was performed on the six passages that had already been identified by visual inspection.

#### 4.2.2 Kymograph-based Target Score analysis

The kymograph representation provided a compact spatiotemporal visualization of the complete image sequence and enabled coherent target-passage patterns to be visualized more efficiently than by direct frame-by-frame inspection of long recorded movies. The appearance of coherent trajectories and transient bands in detectable cases is consistent with the expected spatial and temporal evolution of a moving target through the imaging window, whereas weaker and fragmented patterns in non-detectable cases indicate reduced spatiotemporal coherence relative to the surrounding background.

The kymograph-derived features were combined into the composite Target Score, $S_{\mathrm{CTC}}\left(t\right)$, which was developed to serve two complementary purposes: automated classification of complete experiments and temporal guidance of visual inspection.

For automated classification, the final Target Score threshold of $S_{\mathrm{CTC}}=4.0$ was used to assign each experiment as algorithm-positive or algorithm-negative. The algorithm agreed with the operator-identified passage status in nine of the eleven experimental conditions, with four operator-positive conditions classified as algorithm-positive and five operator-negative conditions classified as algorithm-negative. Two operator-positive conditions were classified as algorithm-negative, and no operator-negative conditions were classified as algorithm-positive, corresponding to an operator-referenced sensitivity of 0.667, specificity of 1.000, precision of 1.000, accuracy of 0.818, and F1 score of 0.800. The ROC analysis yielded an operator-referenced AUC of 0.733. These results quantify agreement with the operator-identified passage status, while also showing that some visually identified passages remained below the automatic detection threshold.

The quantitative classification results and parameter sensitivity (**Supplementary Materials B**) should be interpreted in the context of the limited number of experimental cases. The Target Score parameters were selected using the available 11 experimental conditions, and the same dataset was used to assess the resulting classification performance; no independent training and test datasets were used. Therefore, the reported metrics represent operator-referenced, in-sample performance under the investigated experimental conditions and should not be interpreted as estimates of generalization to unseen samples or experimental settings. Larger datasets with independent validation experiments will be required to establish the generalizability and robustness of the automated classification framework.

Within the operator-referenced classification framework, the absence of ON–AP outcomes at the selected threshold resulted in high specificity and precision, but came at the cost of reduced sensitivity. This trade-off is relevant for transient CTC detection, where weak or partially obscured signals may be difficult to distinguish from background fluctuations, particularly in blood. The selected threshold therefore represents a balance between rejecting background fluctuations and retaining weaker candidate events rather than a universal detection threshold. Given the limited number of experimental cases, the reported classification metrics should be regarded as proof-of-concept performance rather than estimates of clinical diagnostic performance.

As a second use of the same Target Score, temporal candidate windows were generated as a tool to guide visual inspection of the complete recordings in a complementary way. Five of six operator-defined passages were captured by at least one guidance window, corresponding to a passage hit rate of 83.3%. For the captured passages, the mean passage coverage was 92.3% and the median temporal IoU was 0.889. The merged candidate windows represented only 8.21% of the total recorded duration, corresponding to a 91.79% reduction in the temporal search space.

These results indicate that the Target Score can substantially reduce the temporal extent of recordings requiring focused inspection while retaining candidate intervals associated with most visually identified passages. Importantly, candidate intervals that remained below the automatic classification threshold were retained as guidance windows rather than discarded. This allows the same framework to support a more sensitive visual-inspection workflow than would be obtained from binary automatic classification alone.

The difference between passage coverage and temporal IoU further reflects the purpose of temporal guidance. A guidance window may cover most or all of a true passage while extending beyond its manually defined boundaries, resulting in high coverage but lower IoU. Such windows can nevertheless be useful because the objective of temporal guidance is to direct the operator to the relevant temporal region rather than to reproduce the exact temporal boundaries of the passage. In the present study, the recordings were independently inspected to establish the operator-identified passage reference before the temporal-guidance analysis was applied. In a future workflow, the guidance could instead be applied prospectively from the beginning of long recordings to direct the operator toward candidate intervals during the initial inspection, thereby reducing the need for exhaustive frame-by-frame review.

Although this framework provides an automatic binary classification, its intended use at the present stage of development is semi-automated. The automatic Target Score classification can rapidly screen complete recordings, while sub-threshold candidate windows and automatically flagged events can be reviewed using the original image sequence and kymograph. This human-in-the-loop approach is particularly relevant for the current proof-of-concept stage, where visually confirmed events can remain below the automatic threshold and candidate score peaks may not always correspond to confirmed CTC passages.

### 4.3 Limitations and future perspectives

Several limitations should be considered. The experiments were performed in a phantom environment rather than *in vivo* tissue. Although the lower-lip phantom incorporated vessel-mimicking channels, tissue-mimicking material, and donated human blood, the experimental geometry and optical-acoustic environment remain simplified compared with physiological circulation. In particular, the present study did not reproduce the blood vessel curvature, branching, multilumen geometry, pulsatility, and heterogeneous tissue environment encountered *in vivo*. In a clinical setting, such tissue heterogeneity and more complex vessel geometries could introduce spatial variations in optical fluence and acoustic background, potentially altering the photoacoustic signal from a passing target and reducing detection robustness. In addition, physiological movements associated with respiration, speech, swallowing, or other facial movements could introduce temporal fluctuations and motion artifacts that may interfere with the kymograph-based analysis. These effects were not represented in the present static phantom and should be incorporated in future anatomically realistic and dynamically moving models.

The number of investigated experimental conditions was also limited. Each experimental condition was measured only once because of the limited availability of donated blood and cells, precluding an assessment of between-measurement variability. The six passage events used for the SBR/CNR ablation analysis provide a paired proof-of-concept assessment of SVD filtering, but they are insufficient to establish generalizable population-level performance. Similarly, the observed relationships between target size, pigmentation, and detectability do not establish a general minimum detectable cluster size or melanin content, because these factors were not systematically varied independently. A systematic study with controlled variation of cluster size and pigmentation would be required to establish such detection limits and to quantify the relationship between melanin content and photoacoustic signal amplitude.

The blood experiments further indicate that endogenous background signals remain an important challenge. Future experiments should therefore investigate more physiologically representative flow conditions, including pulsatile flow and more complex or curved vessel geometries, before progression toward in-vivo validation. Such experiments would also help determine whether the spatial coherence exploited by the kymograph and Target Score remains sufficiently robust under more realistic motion and background conditions.

Another limitation is that the proposed framework still relies on visual confirmation by the operator. Although the Target Score provides temporal guidance and substantially reduces the fraction of the recording requiring focused inspection, it does not yet constitute a fully autonomous CTC detector. Future work should therefore investigate automated trajectory extraction, larger and more diverse datasets, and machine-learning-based classification of candidate temporal events.

An additional consideration for translation to continuous labial monitoring is optical and thermal safety. The maximum single-pulse fluence used in the present system was 2.42 mJ/cm^2^ at 700 nm, which is well below the commonly cited skin maximum permissible exposure (MPE) of 20 mJ/cm^2^ at this wavelength [6]. However, this value should be regarded only as a conservative reference, since current laser-safety guidelines primarily provide exposure limits for ocular and skin tissues and do not establish a dedicated MPE for oral or labial mucosa under repetitive photoacoustic imaging conditions. The proposed 100-Hz operation therefore requires assessment of cumulative optical exposure and tissue temperature during prolonged illumination. Oral photobiomodulation studies demonstrate that substantially higher light doses can be applied in oral applications, but these therapeutic exposures cannot be directly equated with photoacoustic imaging conditions [35]. Dedicated mucosa-specific dosimetry and thermal measurements will consequently be required before continuous human use. From an engineering perspective, a future mouthguard-integrated implementation would additionally require miniaturization of the laser-delivery and ultrasound-detection components, stable optical and acoustic coupling, optical shielding, heat management, and low-power operation.

Finally, the present study used melanoma-cell-like targets with different levels of pigmentation as a proof-of-concept model. Future studies should systematically investigate different melanoma-cell types, melanin concentrations, target sizes, and target concentrations to determine the conditions under which photoacoustic detection is feasible. Although melanin-rich targets were clearly distinguishable from the blood background at the 700-nm excitation wavelength in the present experiments, multi-wavelength measurements could provide additional spectral information to improve discrimination between melanin and hemoglobin under more heterogeneous physiological conditions. Establishing these limits, together with validation at lower, clinically relevant target concentrations and under increasingly physiological flow and tissue conditions, will be important before assessing the potential of this approach for continuous or repeated CTC monitoring in clinical settings.

## 5 Conclusion

In this study, a proof-of-concept photoacoustic framework was developed to investigate the visualization and temporal characterization of melanoma-cell-like targets flowing through vessel-mimicking channels in a lower-lip phantom. SVD-enhanced photoacoustic imaging and kymograph-based analysis enabled transient target-passage events to be visualized under both PBS and blood-containing conditions. Target detectability depended on target size, pigmentation, and surrounding medium, with larger and more highly pigmented targets generally exhibiting greater visibility, while blood introduced additional background fluctuations that reduced visual contrast. The composite kymograph-derived Target Score provided two complementary functions: automated classification and temporal guidance of visual inspection. Operator-referenced automated classification at the selected threshold yielded an F1 score of 0.800 and an AUC of 0.733, while temporal guidance captured 83.3% of visually identified passages and reduced the temporal search space by 91.79%. These classification metrics represent in-sample performance relative to operator-identified passage status. Quantitative SBR/CNR analysis further showed that SVD filtering consistently improved signal–background separation across all investigated passage events. At this proof-of-concept stage, the framework is therefore best considered a semi-automated approach in which automatic scoring and temporal guidance assist, rather than replace, visual confirmation. Further validation with larger datasets, systematically varied target size and melanin content, and more physiologically representative flow and tissue conditions will be important to further establish the potential of the approach for CTC monitoring and future CTC-informed cancer management.

## CRediT authorship contribution statement

**Ashkan Ghanbarzadeh-Dagheyan:** Writing – original draft, Visualization, Supervision, Methodology, Funding acquisition, Conceptualization. **Elahe Rastegar Pashaki:** Writing – review & editing, Writing – original draft, Software, Methodology, Formal analysis, Data curation. **Kirsten Pondman:** Writing – review & editing, Validation, Resources, Investigation, Data curation.

## Declaration of competing interest

The authors declare the following financial interests/personal relationships which may be considered as potential competing interests: Ashkan Ghanbarzadeh-Dagheyan reports financial support was provided by the Dutch Research Council (NWO) through the Open Mind Programme (Grant No. 17313) and the Veni Programme (Grant No. 19011). If there are other authors, they declare that they have no known competing financial interests or personal relationships that could have appeared to influence the work reported in this paper.

## Data Availability

Data will be made available on request.

## Appendix A Supplementary data

MMC S1
