# Supplementary material for: Lower lip as a photoacoustic imaging window for detection of CTC clusters: A feasibility phantom study
Source: Photoacoustics. 2026 Oct 8;52:100896. doi: 10.1016/j.pacs.2026.100896 (PMC13717880; doi:10.1016/j.pacs.2026.100896)
Supplement: Supplementary file 1 [file mmc1.pdf]

Lower Lip as a Photoacoustic Imaging Window for Detection of Circulating Tumor Cells (CTCs): A Phantom Study

Supplementary Materials

Ashkan Ghanbarzadeh-Dagheyan, Elahe Rastegar Pashaki, Kirsten Pondman

A Quantitative Signal–Background Analysis

A.1 Purpose of the analysis

To provide quantitative characterization of the signal–background separation of visually identified melanoma-cell passage events, an additional signal-to-background ratio (SBR) and contrast-to-noise ratio (CNR) analysis was performed on the recorded photoacoustic image sequences.

The analysis was performed for the six experimental conditions in which the operator identified a CTC passage and defined both the beginning and end of the passage using the visual ground-truth annotation tool. The analysis was performed independently of the automatic binary classification reported in the main manuscript. Its purpose was to quantify the relative separation between temporally varying signal regions and the surrounding background, rather than to establish an additional detection threshold.

A.2 Passage interval definition

For each visually identified passage, the operator-defined start and end frames were obtained from the visual ground-truth annotations. To reduce the influence of uncertainty at the boundaries of the manually selected passage interval, the first and last annotated frames were excluded from the quantitative analysis. The resulting core passage interval was defined as

$$\mathcal{F}_{\text{core}} = \{f_{\text{start}} + 1, \ldots, f_{\text{end}} - 1\}. \tag{1}$$

This exclusion was applied consistently to all analyzed passages. It was not used to redefine the visual ground truth, but rather to avoid including frames at the transition into and out of the passage, where the target may only partially occupy the imaging field.

A.3 Temporal-variation-based signal mask

The photoacoustic image stack and the predefined imaging ROI used by the processing pipeline were loaded for each experiment. For each pixel within the ROI, the temporal standard deviation was calculated over the core passage interval:

$$\sigma_t(x, y) = \text{std}_{f \in \mathcal{F}_{\text{core}}} \left[I(x, y, f) \right], \tag{2}$$

where $I(x, y, f)$ denotes the photoacoustic intensity at pixel (x, y) and frame f.

Because a CTC passage represents a transient event, pixels associated with the moving target and other temporally varying structures are expected to exhibit greater temporal variation than

relatively stationary regions. The temporal standard deviation was therefore used to define a candidate signal mask without manually selecting the target location.

For each experiment, the distribution of σ_t within the predefined ROI was calculated. The primary signal mask was defined using the upper 5% of temporal variation, corresponding to the 95th percentile:

$$T_{95} = P_{95}\left(\sigma_t(x, y) \mid (x, y) \in \text{ROI}\right), \tag{3}$$

and

$$M_{\text{sig}}(x, y) = \begin{cases} 1, & \sigma_t(x, y) \geq T_{95}, \\ 0, & \sigma_t(x, y) < T_{95}, \end{cases} \qquad (x, y) \in \text{ROI}. \tag{4}$$

The remaining pixels within the predefined ROI were assigned to the background mask:

$$M_{\text{bg}}(x, y) = \text{ROI}(x, y) \wedge \neg M_{\text{sig}}(x, y). \tag{5}$$

Thus, the signal mask represents the most temporally varying 5% of the predefined ROI during the passage interval. The mask is a temporal-variation-based candidate signal region and may therefore include other temporally varying structures in addition to the CTC itself. It was consequently used for relative signal–background characterization rather than as a segmentation of the CTC.

A.4 Signal-to-background ratio

For each frame within the core passage interval, the mean intensity within the signal and background masks was calculated as

$$\mu_{\text{sig}}(f) = \text{mean}\left[I(x, y, f) \mid M_{\text{sig}}(x, y) = 1\right], \tag{6}$$

and

$$\mu_{\text{bg}}(f) = \text{mean}\left[I(x, y, f) \mid M_{\text{bg}}(x, y) = 1\right]. \tag{7}$$

The signal-to-background ratio was then calculated for each frame as

$$\text{SBR}(f) = \frac{\mu_{\text{sig}}(f)}{\mu_{\text{bg}}(f)}. \tag{8}$$

An SBR greater than unity indicates that the temporally varying candidate signal region has a higher mean photoacoustic intensity than the corresponding background region. Higher SBR values therefore indicate greater relative signal–background separation.

A.5 Contrast-to-noise ratio

The standard deviation of the background pixels was calculated for each frame:

$$\sigma_{\text{bg}}(f) = \text{std}\left[I(x, y, f) \mid M_{\text{bg}}(x, y) = 1\right]. \tag{9}$$

The contrast-to-noise ratio was subsequently calculated as

$$\text{CNR}(f) = \frac{|\mu_{\text{sig}}(f) - \mu_{\text{bg}}(f)|}{\sigma_{\text{bg}}(f)}. \tag{10}$$

Higher CNR values indicate greater separation between the candidate signal and background relative to the background intensity variability. Unlike SBR, CNR is not referenced to unity.

A.6 Passage-level summary

Because the target moves through the imaging field, SBR and CNR vary over the duration of a passage. For each experiment, the frame-wise SBR and CNR values were therefore summarized using their median values over the complete core passage interval:

$$\mathrm{SBR}_{\mathrm{passage}} = \mathrm{median}_{f\in\mathcal{F}_{\mathrm{core}}}\left[\mathrm{SBR}(f)\right], \tag{11}$$

$$\mathrm{CNR}_{\mathrm{passage}} = \mathrm{median}_{f\in\mathcal{F}_{\mathrm{core}}}\left[\mathrm{CNR}(f)\right]. \tag{12}$$

The median was selected to characterize the typical signal–background separation during the passage while reducing the influence of individual peak frames as the target enters or leaves the imaging window.

Only experiments with an operator-defined CTC passage interval were included. Experiments for which the operator did not identify a passage, and for which the ground-truth start and end frames were therefore undefined, were not assigned SBR or CNR values.

A.7 SVD Ablation Analysis

A.8 Comparison with unfiltered image sequences

To quantify the contribution of SVD preprocessing to signal–background separation, the same SBR/CNR analysis was repeated using the corresponding image sequences without SVD filtering.

The SVD and No-SVD analyses were performed as a paired comparison. For every experiment, the following were kept identical:

- the operator-defined passage interval;
- exclusion of the first and last annotated frames;
- the predefined imaging ROI;
- the temporal-variation percentile used to define the signal mask;
- the SBR definition; and
- the CNR definition.

The only difference between the two analyses was the image stack used as input: the SVD-filtered stack or the corresponding unfiltered stack.

For each experiment, the difference between SVD and No-SVD processing was calculated as

$$\Delta\mathrm{SBR} = \mathrm{SBR}_{\mathrm{SVD}} - \mathrm{SBR}_{\mathrm{NoSVD}}, \tag{13}$$

and

$$\Delta\mathrm{CNR} = \mathrm{CNR}_{\mathrm{SVD}} - \mathrm{CNR}_{\mathrm{NoSVD}}. \tag{14}$$

The corresponding relative changes were calculated as

$$\Delta \mathrm{SBR}_{\%} = 100 \frac{\mathrm{SBR}_{\mathrm{SVD}} - \mathrm{SBR}_{\mathrm{NoSVD}}}{\mathrm{SBR}_{\mathrm{NoSVD}}}, \tag{15}$$

and

$$\Delta \mathrm{CNR}_{\%} = 100 \frac{\mathrm{CNR}_{\mathrm{SVD}} - \mathrm{CNR}_{\mathrm{NoSVD}}}{\mathrm{CNR}_{\mathrm{NoSVD}}}. \tag{16}$$

Because only six paired experiments were available, the paired differences were additionally evaluated using the two-sided Wilcoxon signed-rank test. The statistical analysis was considered exploratory and was used to quantify the consistency of the paired SVD versus No-SVD differences rather than to establish population-level performance.

A.9 Results of the SVD ablation

SVD preprocessing increased both SBR and CNR in all six visually identified passage events (Table A1). The median case-level SBR increased from 1.358 without SVD to 2.701 following SVD filtering, corresponding to a 98.9% increase. The median case-level CNR increased from 0.351 to 2.088, corresponding to a 495.5% increase.

The improvement was consistent across all six paired experiments. SBR was higher following SVD processing in all six cases, while CNR was also higher in all six cases. The largest relative changes were observed for cases with low No-SVD CNR values. Two-sided paired Wilcoxon signed-rank tests yielded $p = 0.03125$ for both SBR and CNR.

Table A1: Paired comparison of signal-to-background ratio (SBR) and contrast-to-noise ratio (CNR) for SVD-filtered and unfiltered photoacoustic image sequences. The same operator-defined passage intervals, imaging ROIs, temporal-variation signal masks, and SBR/CNR definitions were used for both conditions. Values represent median SBR and CNR over the core passage interval.

**Experiment**	**SBR, No SVD**	**SBR, SVD**	**CNR, No SVD**	**CNR, SVD**
Mel624+α-MSH, 500 μm, blood	1.270	1.781	0.280	1.114
Mel624+α-MSH, 500 μm, PBS	1.215	3.025	0.200	2.752
Mel624, 500 μm, PBS	1.574	2.744	0.588	2.136
HS936.T, 1000 μm, PBS	1.335	4.838	0.320	2.721
HS936.T, 500 μm, PBS	1.642	2.658	0.637	2.040
HS936.T, 700 μm, PBS	1.381	2.136	0.382	1.740
**Median across experiments**	**1.358**	**2.701**	**0.351**	**2.088**

A.10 Sensitivity to the temporal-variation percentile

To assess whether the observed SVD advantage depended strongly on the selected temporal-variation threshold, the analysis was repeated using the 90th, 95th, and 99th percentiles of the temporal standard deviation within the ROI. These thresholds corresponded approximately to signal-mask fractions of 10%, 5%, and 1%, respectively.

SVD processing produced higher SBR and CNR than No-SVD processing for all six experiments at all three tested percentile values. Thus, the direction of the SVD effect was unchanged across the tested signal-mask definitions.

Table A2: Sensitivity of the paired SVD versus No-SVD comparison to the percentile used to define the temporal-variation-based signal mask. Values represent median passage-level SBR and CNR.

**Experiment**	**90th**		**95th**		**99th**	
	SBR	CNR	SBR	CNR	SBR	CNR
Mel624+α-MSH, 500 μm, blood	1.771	1.057	1.781	1.114	1.877	1.192
Mel624+α-MSH, 500 μm, PBS	2.584	2.541	3.025	2.752	3.339	1.841
Mel624, 500 μm, PBS	2.494	2.079	2.744	2.136	3.139	2.273
HS936.T, 1000 μm, PBS	4.502	3.868	4.838	2.721	4.695	2.366
HS936.T, 500 μm, PBS	2.413	1.886	2.658	2.040	3.772	2.708
HS936.T, 700 μm, PBS	1.979	1.566	2.136	1.740	2.103	1.469

Across the tested percentiles, the SVD-filtered sequences consistently exhibited greater signal–background separation than the corresponding No-SVD sequences. The consistent direction of the paired differences indicates that the observed improvement was not dependent on a single choice of temporal-variation percentile.

B Sensitivity analysis of Target Score weights and decision threshold

To assess the sensitivity of the automated CTC classification to the parameters of the composite Target Score, we systematically varied the three component weights and the final decision threshold. The Dark, Bright, and Direction weights were varied in increments of 0.05, subject to the constraint that their sum was equal to one. The Target Score threshold was varied from 0.5 to 5.5 in increments of 0.25. This resulted in a total of 4,809 combinations of weights and decision thresholds. For each parameter combination, the resulting binary classifications were compared with the operator-defined visual ground truth, and the F1 score was calculated.

Figure B1 summarizes the sensitivity analysis. Figure B1(a) shows the maximum F1 score obtained across all weight combinations for each decision threshold. The highest F1 score of 0.80 was obtained over a range of thresholds, indicating that the classification performance was not strongly dependent on a single threshold value. Figure B1(b) shows the dependence of the F1 score on the Dark and Bright weights at the selected threshold of 4.0, with the Direction weight determined by the constraint $w_{\text{direction}} = 1 - w_{\text{dark}} - w_{\text{bright}}$. The parameter combination used in the main analysis, $w_{\text{dark}} = 0.60$, $w_{\text{bright}} = 0.15$, and $w_{\text{direction}} = 0.25$, yielded an F1 score of 0.80, equal to the maximum F1 score observed across the complete parameter sweep. The selected parameter set achieved the maximum F1 score observed in the parameter sweep (F1 = 0.80). Importantly, this maximum was shared by several parameter combinations, indicating a plateau of equivalent classification performance rather than a uniquely optimal solution. The selected configuration retained a substantial contribution from the directionality component ($w_{\text{direction}} = 0.25$), while achieving the same maximum classification performance as configurations with lower or negligible directionality weighting.

Overall, the sensitivity analysis indicates that the observed classification performance was relatively stable to moderate changes in the Target Score weights and decision threshold within the investigated parameter range. The selected parameters were therefore retained for the primary analysis.

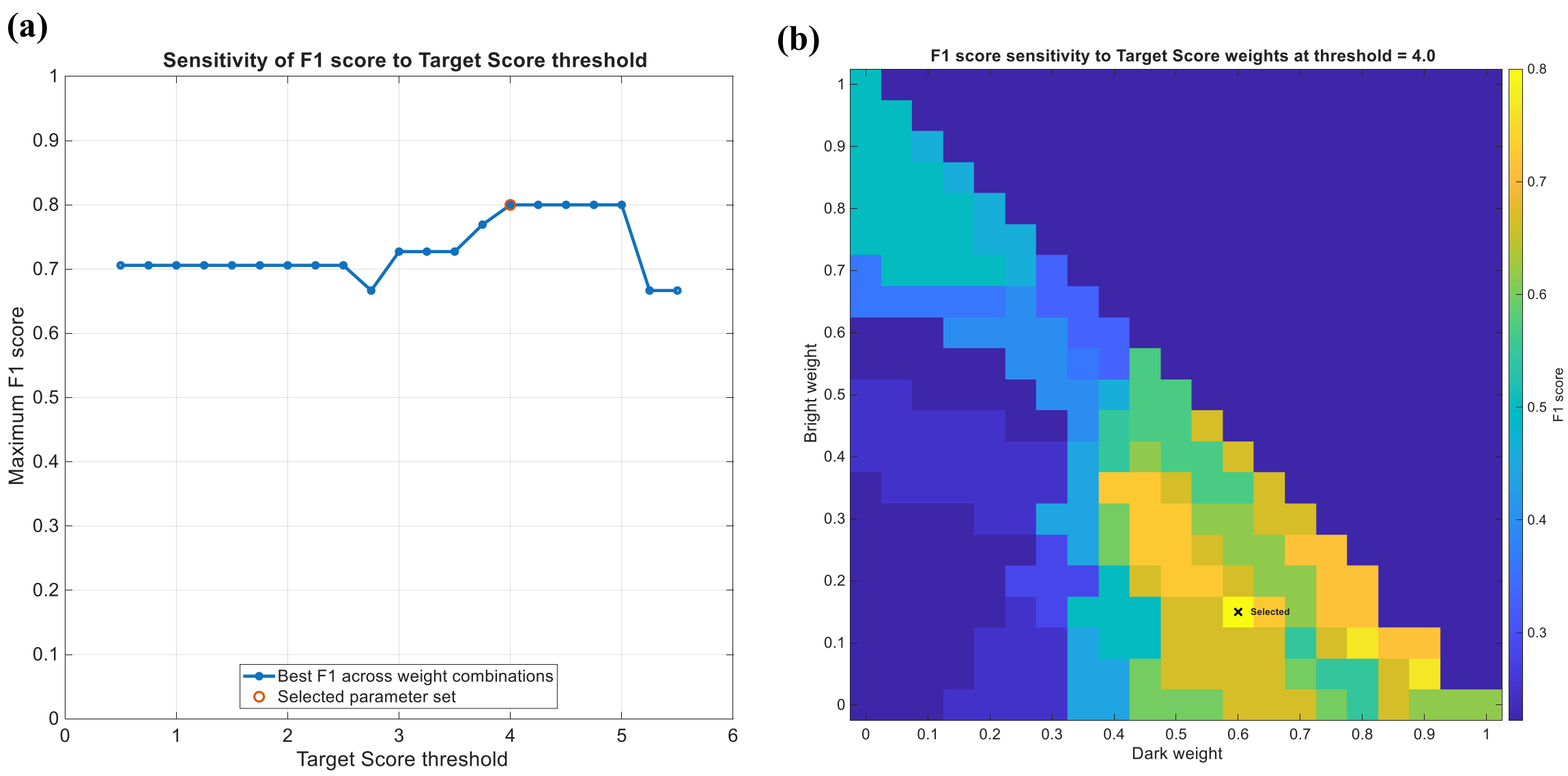

Figure B1: Sensitivity of the automated CTC classification to the Target Score parameters. (a) Maximum F1 score obtained across all tested combinations of the Dark, Bright, and Direction weights as a function of the Target Score decision threshold. (b) F1 score as a function of the Dark and Bright weights at the selected decision threshold of 4.0, with the Direction weight constrained such that the three weights sum to one. The black cross indicates the parameter combination used in the main analysis ($w_{\text{dark}} = 0.60$, $w_{\text{bright}} = 0.15$, $w_{\text{direction}} = 0.25$).
